# Ecological niche contributes to the persistence of the western × glaucous‐winged gull hybrid zone

**DOI:** 10.1002/ece3.11678

**Published:** 2024-07-11

**Authors:** Xuewen Geng, Jeremy Summers, Nancy Chen

**Affiliations:** ^1^ Department of Biology University of Rochester Rochester New York USA

**Keywords:** bounded superiority model, ecological niche modeling, hybrid zone, niche identity test

## Abstract

Hybrid zones occur in nature when populations with limited reproductive barriers overlap in space. Many hybrid zones persist over time, and different models have been proposed to explain how selection can maintain hybrid zone stability. More empirical studies are needed to elucidate the role of ecological adaptation in maintaining stable hybrid zones. Here, we investigated the role of exogenous factors in maintaining a hybrid zone between western gulls (*Larus occidentalis*) and glaucous‐winged gulls (*L. glaucescens*). We used ecological niche models (ENMs) and niche similarity tests to quantify and examine the ecological niches of western gulls, glaucous‐winged gulls, and their hybrids. We found evidence of niche divergence between all three groups. Our results support the bounded superiority model, providing further evidence that exogenous selection favoring hybrids may be an important factor in maintaining this stable hybrid zone.

## Graphical abstract

In this study, we investigated the role of exogenous factors in maintaining a hybrid zone between western gulls (*Larus occidentalis*) and glaucous‐winged gulls (*L. glaucescens*). We used ecological niche models (ENMs) and niche similarity tests and found evidence of niche divergence between all three groups. Our results best support the bounded superiority model, providing further evidence that exogenous selection favoring hybrids may be an important factor in maintaining this stable hybrid zone.

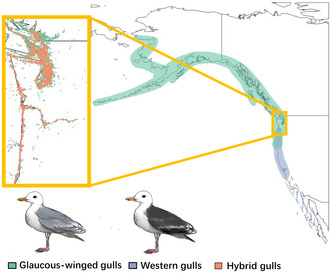

## 1 INTRODUCTION

Hybridization is widely observed among related species, and the areas where lineages overlap (hybrid zones) offer a unique opportunity to investigate the role of ecology in speciation (Abbott et al., 2013; Barton & Hewitt, 1985). In many cases, there is a balance between selection and gene flow within a hybrid zone, resulting in a stable hybrid zone over time (Barton, 1979; Moore, 1977). Both environmental variation and geographical barriers within a hybrid zone can facilitate the physical separation of the parental populations, potentially reinforcing existing levels of reproductive isolation and population divergence. At the same time, there are opportunities for hybrids with unique trait combinations or intermediate features to secure ecological niches different from their parental species along an environmental gradient (Buggs, 2007; Endler, 1977; Harrison, 1993; Taylor et al., 2015).

Previous studies have proposed multiple models to explain hybrid zone stability. These models can be classified based on whether the selective forces acting on hybrids are endogenous or exogenous (Moore, 1977). Endogenous selection refers to selection due to genetic incompatibilities independent of the environment, while exogenous selection refers to the differential selection of hybrids depending on the environment (Barton & Hewitt, 1985). The tension zone model proposes that hybrid zones are maintained by an equilibrium between the dispersal of parental species into the hybrid zone and endogenous selection against hybrids (Key, 1968; Moore, 1977). The geographical selection‐gradient model also states that hybrid zone stability is maintained by a balance between the dispersal of parental species and selection against hybrids, but here selection against hybrids is exogenous (Barton & Hewitt, 1985; Moore, 1977). The bounded superiority model argues that exogenous selection favors hybrids over either parental species because hybrids occupy a unique niche within the transitional zone (Moore, 1977). Previous studies have found evidence supporting each of these three models in different hybrid zones (e.g., Culumber et al., 2011; Gay et al., 2008 for the tension zone model; Edwards, 2015 for the geographical selection‐gradient model; De La Torre et al., 2014; Good et al., 2000; Wang et al., 1997 for the bounded superiority model). More studies of hybrid zone stability in other species are necessary to determine which of these models is more prevalent in nature.

One approach to identify the forces maintaining hybrid zone stability is to quantify the role of exogenous factors by comparing the niches of parental species and their hybrids (Swenson, 2006, 2008). Ecological niche models (ENMs) use spatial environmental data and species occurrence localities to predict the possibility of species occurrence across different environments, providing estimates of the realized niche of the focal species (Broennimann et al., 2012; Guisan & Thuiller, 2005). This approach has been used to investigate niche divergence and hybrid zone stability in multiple taxa, such as the hybrid zones between brown lemurs *Eulemur rufifrons* and *E. cinereiceps* (Johnson et al., 2016), tidal marsh birds *Ammodramus caudacutus* and *A. nelsoni* (Walsh et al., 2016), and swordtails *Xiphophorus birchmanni* and *X. malinche* (Culumber et al., 2012).

Here, we used ENMs to investigate the role of environmental variation in maintaining a well‐studied hybrid zone between western gulls (*Larus occidentalis*) and glaucous‐winged gulls (*L. glaucescens*) in the Pacific Northwest region of North America (Bell, 1992, 1996, 1997; Hoffman et al., 1978). Although there is evidence that this hybrid zone has expanded since its discovery (Bell, 1996; Good et al., 2000; Hoffman et al., 1978) and shifted south (Gay et al., 2008), cline analyses do not suggest the species are fusing (Gay et al., 2008). Thus, for the sake of our analysis in this study, we assume that the hybrid zone is stable or persistent over time (as in Megna et al., 2014). Previous studies of this hybrid zone have focused on comparing reproductive success, mating patterns, population structure, and clinal variation of the hybrids and the parental species (Bell, 1992, 1996, 1997; Gay et al., 2008; Good et al., 2000; Hoffman et al., 1978; Megna et al., 2014; Moncrieff et al., 2013). Results of these studies, however, support different models of hybrid zone stability (summarized in Table 1), thus which model best explains the stability of this hybrid zone remains up for debate. No previous studies of this hybrid zone have assessed the significance and contribution of potential differences between the ecological niches of the hybrids and the parental species to hybrid zone stability.

**TABLE 1:** Support for different models of hybrid zone stability from previous published work on the western × glaucous‐winged gull hybrid zone and the present study.

Study focus	Prediction	Model	Outcome
Hybrid reproductive success	(1) Hybrids exhibit lower reproductive success compared to parental species	Tension zone model or geographical selection‐gradient model	No. Reproductive success of hybrids is equal to or higher than that of the parental species (Good et al., 2000; Hoffman et al., 1978; Megna et al., 2014; Moncrieff et al., 2013)
	(2) Hybrids perform equal or better than at least one of the parental species	Bounded superiority model	Yes. Hoffman et al. (1978) and Good et al. (2000) found that hybrids perform better than the parental species. Moncrieff et al. (2013); Megna et al. (2014) found that hybrids and the parental species perform equally
Assortative Mating	(1) Assortative mating exists because hybrids are being selected against: Mating with hybrids results in a decrease in reproductive success	Tension zone model or geographical selection‐gradient model	Maybe. Hoffman et al. (1978), Bell (1997), and Megna et al. (2014) all found evidence of assortative mating. However, Megna et al. (2014) found no decrease in reproductive success for dissimilar pairs, and Good et al. (2000) found no significant correlations between the hybrid indices of male and female gulls
	(2) Preference for hybrid mates	Bounded superiority model	No. No evidence has been found showing a preference for mating with hybrids.
Population density in the hybrid zone	(1) Low density of hybrids within the hybrid zone	Tension zone model or geographical selection‐gradient model	No. Hybrids are prevalent within the hybrid zone (Bell, 1992; Hoffman et al., 1978).
	(2) Higher density of hybrids in the hybrid zone compared to outside the hybrid zone	Bounded superiority model	Yes. Hoffman et al. (1978) and Bell (1992) found that hybrids occur more frequently within the hybrid zone
Cline shape	(1) Clines are sigmoid and narrow in shape and coincident	Tension zone model or geographical selection‐gradient model	Yes. Gay et al. (2008) found sigmoid and narrow clines in phenotypic traits and molecular markers
	(2) Clines have more variable shape and noncoincident	Bounded superiority model	Maybe. Bell (1996) found shallow clines at allozyme markers
Ecological niche	(1) Niche conservatism between all three groups	Tension zone model	Maybe. Range‐breaking tests found no significant geographical boundary between all three groups, but these results could simply indicate a mosaic hybrid zone (this study)
	(2) Niche conservatism between the parental species and the hybrids, and niche divergence between the parental species	Geographical selection‐gradient model	No. There is no evidence of niche conservatism between the parental species and hybrids but niche divergence between the parental species (this study)
	(3) Niche divergence between all three groups	Bounded superiority model	Yes. Niche identity and background similarity tests from ENMs indicate niche divergence among all three groups (this study)

In this study, we tested whether hybrid gulls exhibit a different niche than their parental species and how environmental variation may contribute to the distribution and stability of the hybrid zone. We constructed ENMs, characterized environmental variation associated with the distributions of glaucous‐winged gulls, western gulls, and hybrid gulls, and quantified the differences between the niches occupied by the two parental species and their hybrids. We hypothesized that if there is no niche divergence between the parental species and the hybrids or between the two parental species and the occurrence of hybrids is highly associated with the presence of parental species, then exogenous factors do not contribute to the stability of this hybrid zone, indicating support for the tension zone model. If the hybrid zone is best explained by the geographical selection‐gradient model, we hypothesized that we should observe niche divergence between the two parental species, but not between the hybrids and the parental species, as selection is assumed to be against the hybrids. Instead, if the hybrid zone conforms to the bounded superiority model, we predicted that there should be niche divergence between all three groups.

## 2 MATERIALS AND METHODS

### 2.1 Study system

**FIGURE 1:**
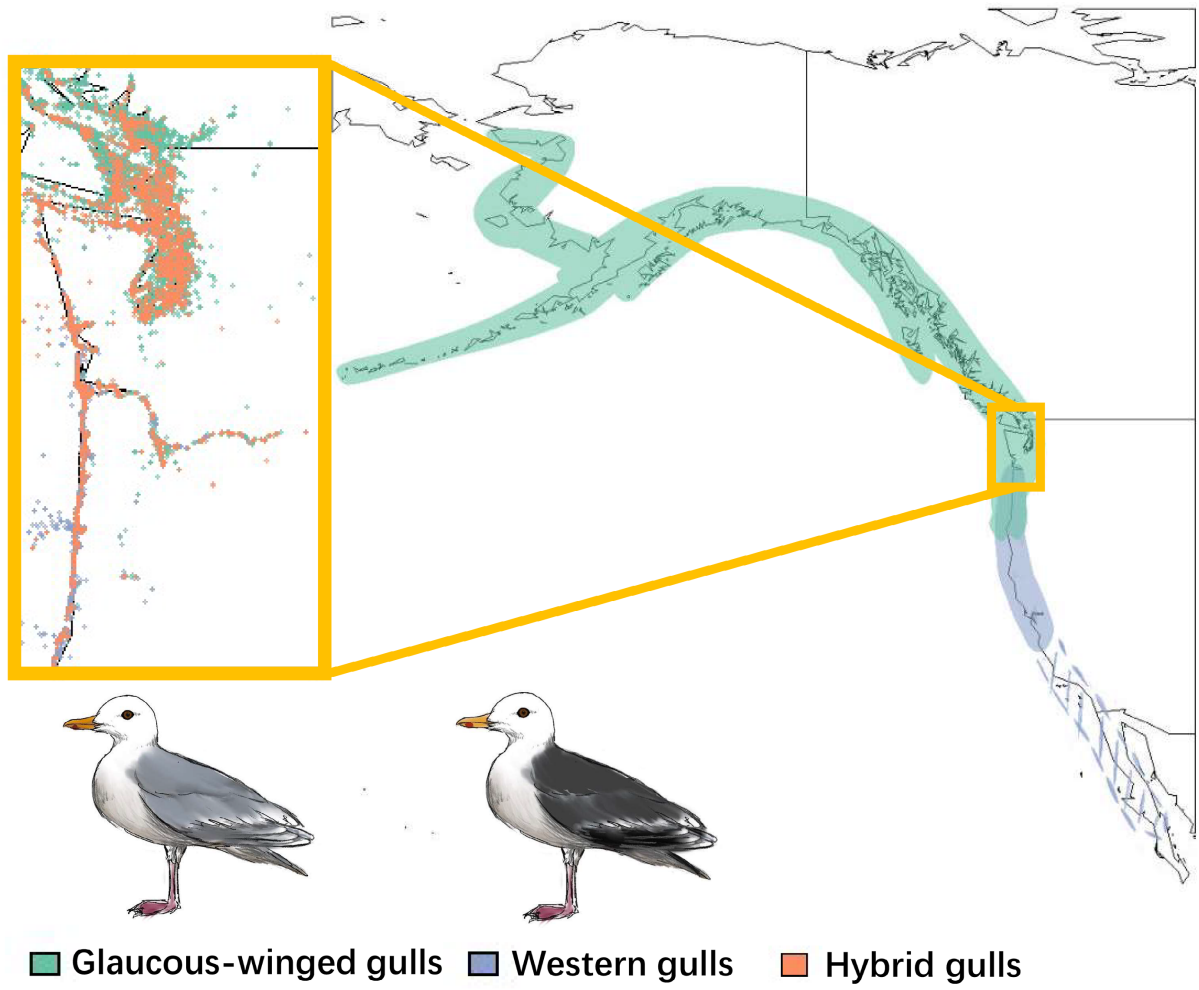
The location of the hybrid zone and the ranges of glaucous‐winged gulls (*Larus glaucescens*; green), western gulls (*Larus occidentalis*; blue), and their hybrids (orange) in North America. There are two recognized subspecies of western gulls: *L. o. occidentalis* is found from Washington to Central California (solid blue), and *L. o. wymani* is found from Central California to Baja California (blue‐dashed lines). The inset shows eBird occurrence data for the two parental species and hybrid individuals in the hybrid zone. Western gulls, glaucous‐winged gulls, and their hybrids can be visually distinguished best by differences in mantle and wingtip plumage color.

We studied the western × glaucous‐winged gull hybrid complex along the western coastline of North America. Western gulls breed from central Baja California north to Washington, and glaucous‐winged gulls breed further north, from Washington to Alaska (Figure 1; Hayward & Verbeek, 2020; Pierotti & Annett, 2020). The hybrid zone is considered to be a narrow ecotone along the Washington coastline (Hoffman et al., 1978; Reagan, 1911). Hybrids between the northern subspecies of western gulls (*Larus occidentalis occidentalis*) and glaucous‐winged gulls were first noted in the early 20th century and are prevalent within the hybrid zone, occurring in higher frequencies than the parental species at some locations (Figure 1; Bell, 1992, 1996; Dawson, 1908; Dawson et al., 1909). These gulls can be distinguished primarily based on their mantle and wingtip plumage color: western gulls have darker gray plumage, glaucous‐winged gulls have lighter gray plumage, and hybrids have an intermediate shade of gray. Other distinguishing traits include iris color, orbital ring color, and beak color (Bell, 1996, 1997; Moncrieff et al., 2013). These three groups can be visually distinguished in the field, allowing us to use citizen science databases for data acquisition.

### 2.2 Species occurrence data

ENMs use both species occurrence data and environmental layers to predict the niche of a species. We obtained occurrence data for the parental species and the hybrids from the citizen science database eBird (Sullivan et al., 2009). eBird is the world's largest citizen science database for bird occurrence records, allowing birders from all around the world to document the distribution, abundance, and identities of the birds they encounter. eBird has a robust review process for ensuring species identity, requiring individuals to submit supporting materials such as photos, audio, field notes, or video evidence for observations of rare or difficult‐to‐identify species, which are then verified by local eBird reviewers (Sullivan et al., 2009). eBird reviewer guidelines classify hybrid western × glaucous‐winged gulls as regular, field‐separable occurrences in Alaska, the Pacific Northwest, and California. Here, we used the eBird Basic Dataset version EBD‐relMay‐2023.

We used the auk package in R to download and filter eBird data (Strimas‐Mackey et al., 2022). Since western gulls and glaucous‐winged gulls are known to be partially migratory, we extracted eBird occurrence data within the breeding season (defined as June to July based on the temporal overlap of their breeding seasons; Annett & Pierotti, 1989; Davis et al., 2015; Murphy et al., 1984; Vermeer et al., 1988). In addition, as hybrid gulls were not frequently reported until 2010, we only included occurrence points recorded between 2010 and 2023. To lessen sampling effort bias, we filtered observations based on survey protocols (Stationary checklists only), duration (<360 min), time of the day (6:00–21:00), and the number of observers (≤10 people) for each observation based on Best Practices for Using eBird Data (Strimas‐Mackey et al., 2022). We also only included checklists marked as complete (ones for which eBird users indicate they recorded every bird they detected) to reduce the impact of taxonomic preferences and bias in detection (Johnston et al., 2021). We set the spatial extent based on the longitudes and latitudes of the occurrence points and to only include the breeding range of the northern western gulls: 114° W–175° W, 31° N–62° N (Bell, 1996, 1997; Hoffman et al., 1978; Figure 1). We then manually removed outliers. Our filtered eBird dataset included 17,061 observations of glaucous‐winged gulls, 9297 observations of western gulls, and 1897 observations of hybrid individuals. The occurrence data span six states across two countries (Canada and the United States), including British Columbia (Canada), Yukon Territory (Canada), California (United States), Oregon (United States), Washington (United States), and Alaska (United States), which fits with current understanding of species ranges for these gulls.

To account for potential sampling effort bias introduced by geographic factors, we performed spatial thinning using the spThin package in R (Aiello‐Lammens et al., 2015). We chose a thinning grid of 0.5 km × 0.5 km because previous studies showed that this grid size efficiently removes redundant records while including spatially valuable data (Steen et al., 2021). We then balanced the sample sizes for each species and the hybrids by randomly sampling records from the thinned dataset. Our final dataset consisted of 600 records for each group (Figure S1).

### 2.3 Environmental data

We included a total of 16 environmental variables in our ENMs. We obtained bioclimatic variables from WorldClim (Fick & Hijmans, 2017), land cover data from the North American Land Change Monitoring System (NALCMS; Homer et al., 2017), elevation data from elevatr package in R (Hollister et al., 2021), and distance from coastline data NOAA National Ocean Service (Stumpf & Kuring, 2009). WorldClim is composed of a set of gridded climate layers with variables related to temperature and precipitation (Fick & Hijmans, 2017). We downloaded the 19 bioclimatic layers from WorldClim using a 2.5‐min spatial resolution. We only included annual measures and excluded the isothermality layer, which is inherently highly correlated with other layers since it is calculated as the mean diurnal range layer divided by the temperature annual range layer. We downloaded land cover data from NALCMS using a 30‐m spatial resolution. This layer includes 19 land cover types that are jointly identified by government agencies from Canada, US, and Mexico. We separated each land cover type into individual layers and calculated their percent coverage in grids using the aggregate() and projectRaster() functions. We downloaded the world elevation map from the elevatr package in R. This package provides a combination of world elevation maps based on publicly accessible remote sensing maps from different countries. We downloaded the distance from the coastline map from NOAA National Ocean Service, which provides a world map of distance from the coastline with an uncertainty of 1 km. Locality grids marked with values <0 represent localities corresponding to the land, and localities marked with values >0 represent localities corresponding to the ocean. We reprojected all environmental layers to match the resolution and extent of the WorldClim dataset.

We then extracted environmental data for each occurrence point from the thinned dataset and tested the correlation between different layers using the vifstep and vifcor tools from the usdm package in R (Naimi et al., 2014). These are two different ways of detecting collinearity using a variance inflation factor (VIF). Vifstep lists variables that yield a higher VIF than the threshold (10), whereas vifcor removes the variable that yields a higher VIF from a pair of variables that has a greater linear correlation than a specified threshold (we used 0.9; Naimi et al., 2014). Using both methods, we removed environmental layers that are highly correlated with other layers. Our final 16 environmental variables were annual mean temperature, mean diurnal range, temperature seasonality, annual precipitation, temporal and subpolar needle leaf forest, temporal and subpolar grassland, wetland, cropland, barren land, urban, snow and ice, temporal and subpolar broad leaf forest, mixed forest, temporal and subpolar shrub land, elevation, and distance to the coastline.

### 2.4 Environmental niche modeling

We used the maximum entropy method in Maxent to construct ENMs. Maxent predicts the suitability of environmental conditions for the species of interest based on species occurrence localities, background points, and environmental layers (Phillips et al., 2006). The final suitability map produced from Maxent represents the probability of occurrence that contains the maximum entropy, or the most spread out distribution (Elith et al., 2011; Phillips et al., 2006). Maxent offers a few advantages that are important to our study: It uses presence‐only data, avoiding potential biases introduced by predicted absence data from complete eBird records (Johnston et al., 2021), and it outputs a continuous probability of suitability raster that allows further comparative analyses between populations (Phillips et al., 2006). The one disadvantage, however, is that Maxent models are sensitive to sampling bias introduced by potential correlations between sampling efforts and specific environmental variables (Johnston et al., 2021; Merow et al., 2013). To correct for potential sampling effort bias introduced by spatial features across our spatial extent, we used the target‐group background method to select background points. The target‐group background method uses the occurrence points of similar species sampled by the same methods within the zone of interest as background datasets to account for sampling bias (Phillips et al., 2009; Vollering et al., 2019). This approach has been proven to be effective in Maxent models (Barber et al., 2022; Phillips & Dudík, 2008). Thus, we downloaded, filtered, and thinned the eBird Basic Dataset (version EBD‐relMay‐2023) for all species using the same protocol we used for the parental species and hybrids. For each model, we extracted 10,000 points from the filtered all‐species dataset within our study extent to serve as our target‐group background points. These background points include checklists both with and without the focal species.

We used the maxent package in R to construct ENMs with our 16 environmental variables, species occurrence localities, and target‐group background points (Jurka, 2012). We cross‐validated each model 10 times with a 20% testing and 80% training percentage to calculate confidence intervals. To evaluate model performance, we calculated the area under the receiver operating curve (AUC) for each model. An AUC value of 0.5 indicates model performance similar to random prediction, and an AUC value of 1 means the model has perfect prediction power. Typically, an AUC value above 0.7 indicates reliable performance (Metz, 1978; Swets, 1988).

We analyzed the importance of each environmental variable using jackknife tests and response curves in the maxent package. The jackknife test independently evaluates the contribution of each environmental variable by creating a series of models that use or exclude each variable in turn and calculating the gain in model performance. Jackknife results for all three species showed similar results for positive regularized training gain, test gain, and AUC value, so we only report training gain results for simplicity. For the response curves, we calculated the mean and confidence intervals over 10 model iterations (Elith et al., 2011; Phillips et al., 2017; Phillips & Dudík, 2008).

To test the accuracy of our eBird models, we tested model transferability using eBird records with verified species identification or breeding records as well as occurrence data from another citizen science database: The North American Breeding Bird Survey (BBS). Model transferability is the ability of one model to accurately predict species occurrence using a dataset other than the training dataset, and is crucial for assessing the validity of ecological niche models (Sequeira et al., 2018; Yates et al., 2018). Please see Appendix S1 for more information on the model transferability tests.

### 2.5 Niche similarity tests

To test for differences in ecological niches between each of the parental species and the hybrids, we performed paired niche identity tests and background similarity tests. We also tested for abrupt biogeographical barriers using blob range‐breaking rests. We performed all tests in the ENMTools package in R (Warren et al., 2021) with two ecological niche indices that are commonly used in quantifying niche overlap: Schoener's *D* and Warren's *I* (Schoener, 1968; Warren et al., 2008). The values of both indices range from zero, indicating no niche overlap, to one, indicating a perfect niche overlap between each pair of populations tested. The niche identity test looks for differences between two ENMs by comparing the ecological niche indices calculated from observed occurrence points to a null distribution of values calculated from randomly assigning data points to two groups (Schoener, 1968; Warren et al., 2008). The symmetric background similarity test assesses differences in ENMs of allopatric groups given the available habitats in the regions in which they occur (Warren et al., 2008). Unlike the niche identity test, the background similarity test generates a null distribution by randomly choosing points from the broad region the groups inhabit and uses a two‐tailed *t*‐test to test for a significant difference between the null distribution and empirical values of Schoener's *D* and Warren's *I*. The blob range‐breaking test uses the same set of indices to check for a distinct boundary between two populations (Glor & Warren, 2011; Warren et al., 2008, 2021). We used 100 replicates to generate null distributions for each test and a significance threshold of 0.05.

## 3 RESULTS

**FIGURE 2:**
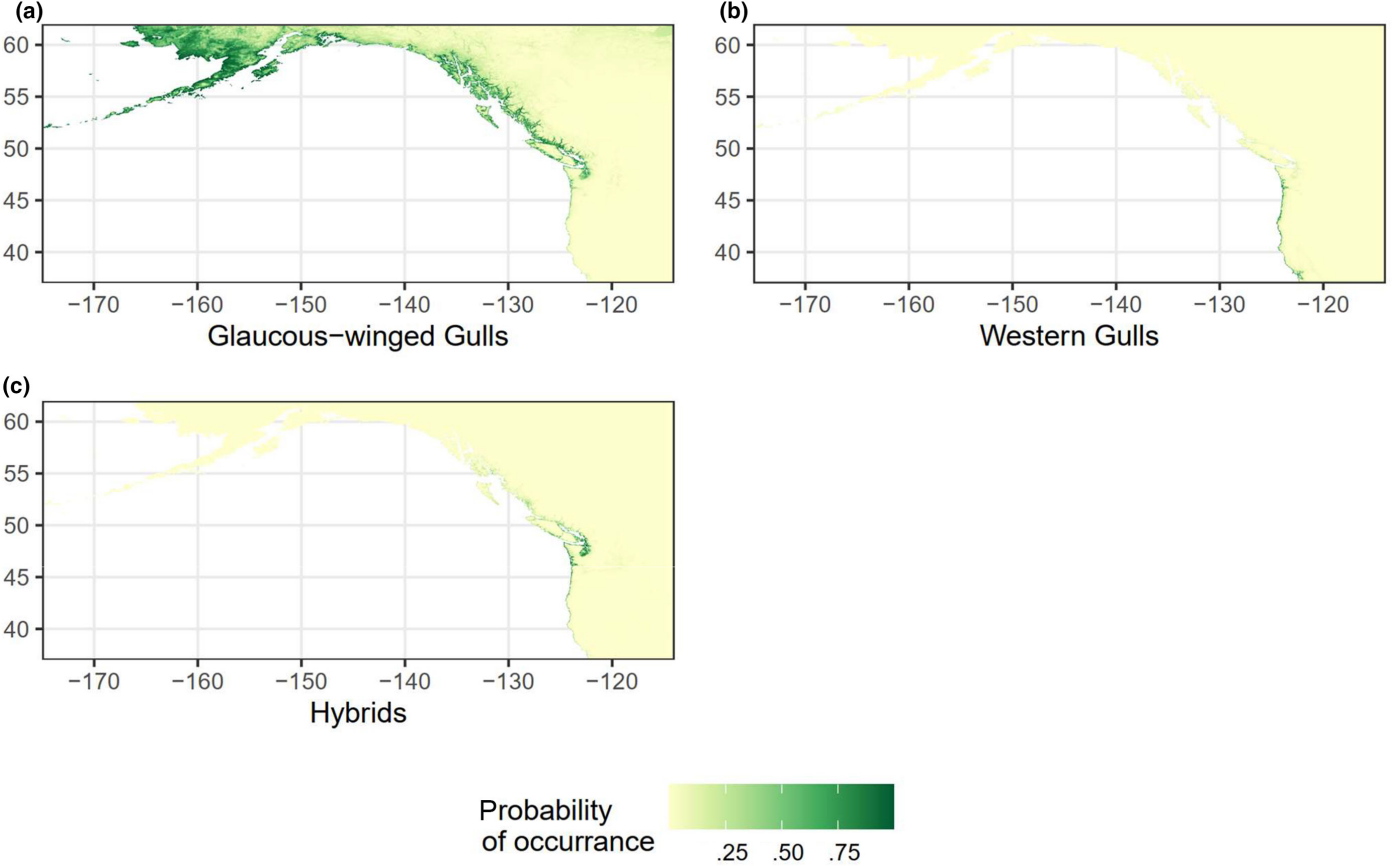
The predicted probability of species occurrence from maxent ENMs of (a) glaucous‐winged gulls, (b) western gulls, and (c) their hybrids. Green represents a higher probability of occurrence and yellow represents a lower probability of occurrence.

To quantify the ecological niches of parental and hybrid gulls, we built ENMs for glaucous‐winged gulls, western gulls, and their hybrids (Figure 2). The mean AUC of our models are all above 0.8 (glaucous‐winged gulls: mean = 0.891, SD = 0.010; western gulls: mean = 0.943, SD = 0.012; hybrids: mean = 0.918, SD = 0.010), and model transferability tests with stricter species identification and breeding status filters all showed high performance (Appendix S1), indicating our models can reliably predict occurrence (Hosmer & Lemeshow, 2000). The predicted species distributions from our ENMs match the known distributions: glaucous‐winged gulls prefer northern habitats and western gulls prefer southern habitats, with an overlap in the middle where the hybrids appear (Figure 1). The hybrid distribution extends beyond the historical recorded hybrid range, but with the highest probabilities of occurrence along the Washington coastline, where most previous studies have found and studied them.

**FIGURE 3:**
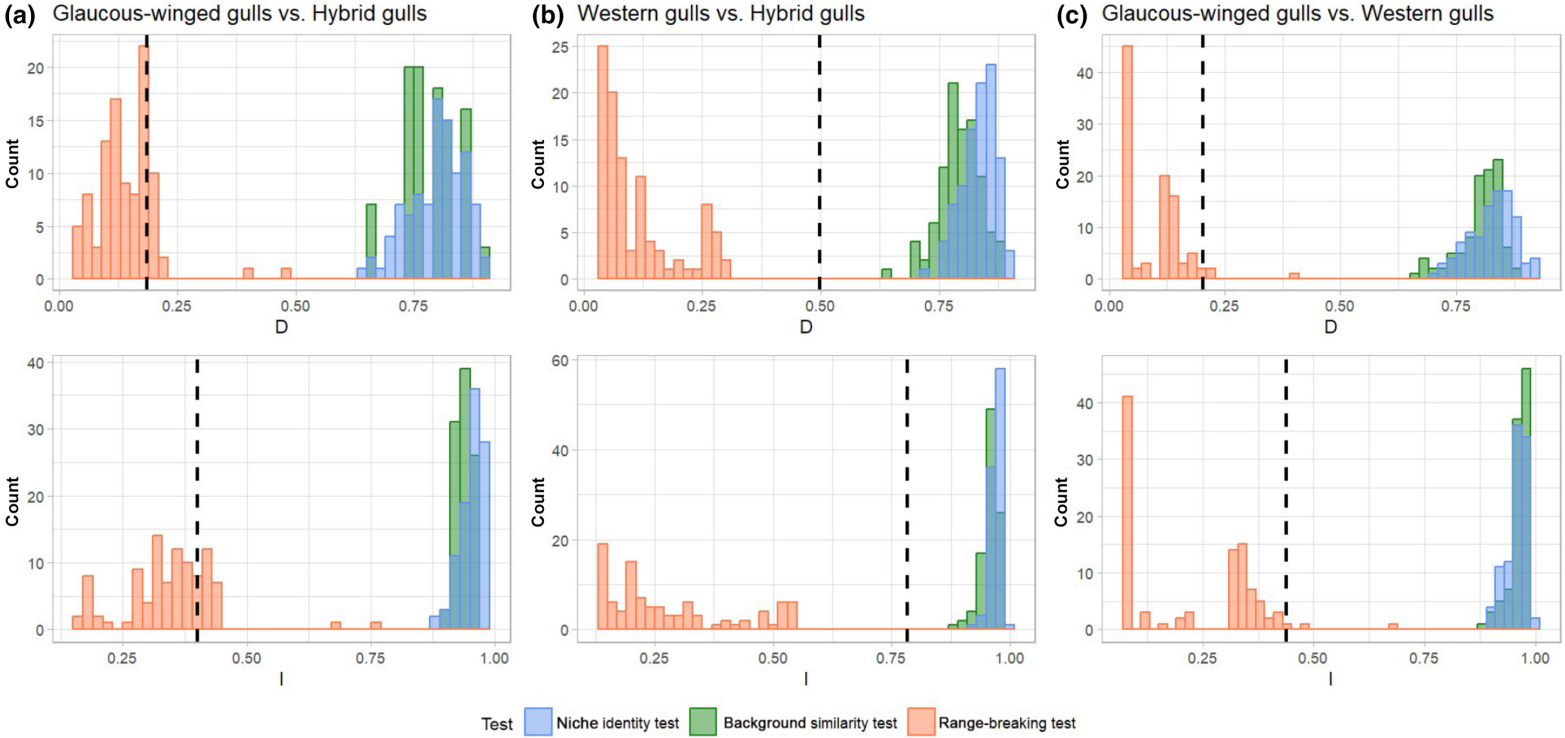
Niche identity (blue), background similarity (green), and range‐breaking (orange) test results between (a) glaucous‐winged gulls and hybrid gulls, (b) western gulls and hybrid gulls, and (c) glaucous‐winged gulls and western gulls. Results for Schoener's *D* are on the top row and Warren's *I* on the bottom. Histograms show the null distribution for each test, and the dotted‐black line is the observed value. All niche identity and background similarity tests show significantly lower‐than‐expected niche indices, indicating significant niche divergence between all pairwise comparisons. The empirical niche indices are not significantly lower than the null distribution of the range‐breaking test for any comparison, so there is no evidence of a biogeographic boundary separating populations.

We calculated the niche indices Schoener's *D* and Warren's *I* (Table 2) and performed niche identity tests and symmetric background similarity tests to test for significant niche divergence between the parental species and the hybrids. Both tests generated distributions of Schoener's *D* and Warren's *I* that are significantly higher than the empirical values for all three pairwise comparisons, suggesting that all three populations have significantly different niches (*p* < .01 for all tests; Figure 3). We performed range‐breaking tests to examine whether there is a distinct boundary between the parents and the hybrids within the hybrid zone and whether physical geological barriers contribute to niche divergence. We found that the empirical indices are not significantly lower than the randomized controls for all three pairwise comparisons in the range‐breaking test (*p* > .89 for all tests; Figure 3), suggesting that there is no abrupt geographical boundary associated with sudden environmental gradients in this hybrid zone.

**TABLE 2:** Niche indices calculated for the niche identity, background similarity, and range‐breaking test between western gulls, glaucous‐winged gulls, and their hybrids.

	Glaucous‐winged gulls vs. hybrid gulls	Western gulls vs. hybrid gulls	Glaucous‐winged gulls vs. Western gulls
Schoener's *D*	0.187	0.497	0.202
Warren's *I*	0.398	0.773	0.429

**FIGURE 4:**
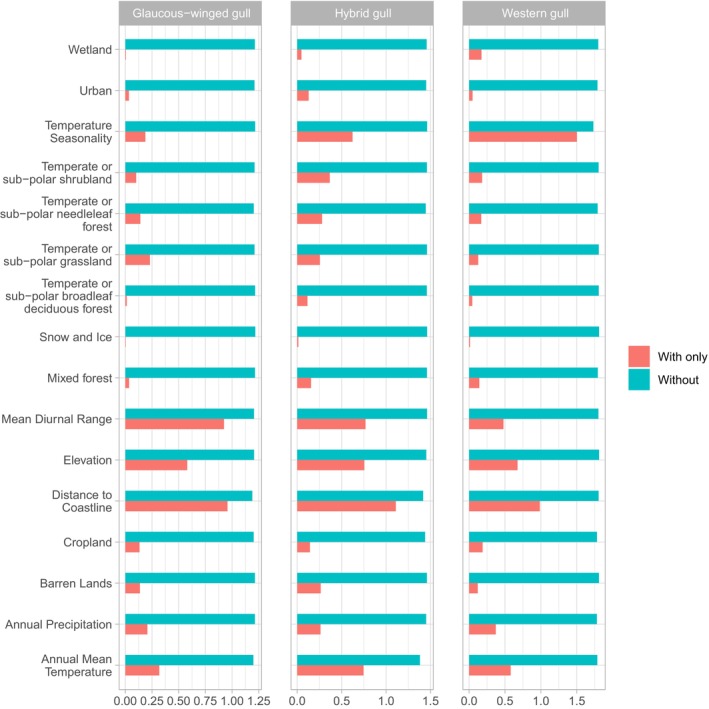
Jackknife tests of variable importance for Maxent models of glaucous‐winged gulls, western gulls, and their hybrids. Teal bars represent the regularized training gain of models excluding the specified variable, and red bars represent the training gain of models that only include the specified variable.

**FIGURE 5:**
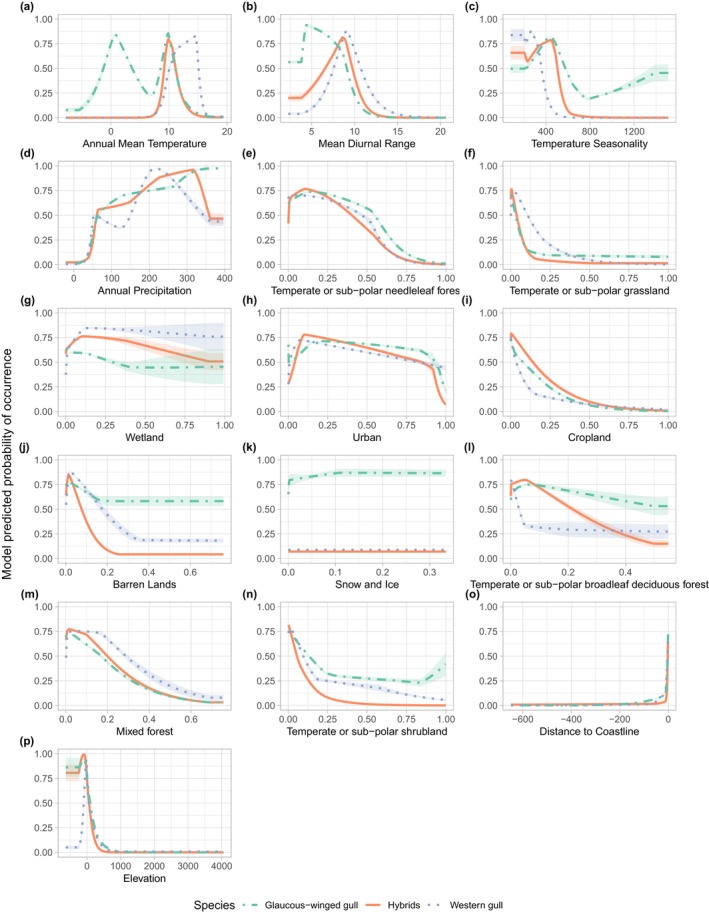
Response curves in relation to (a) annual mean temperature, (b) mean diurnal range, (c) temperature seasonality, (d) annual precipitation, (e) temperate or subpolar needleleaf forest, (f) temperate or subpolar grassland, (g) wetland, (h) urban, (i) cropland, (j) barren lands, (k) snow and ice, (l) temperate or subpolar broadleaf deciduous forest, (m) mixed forest, (n) temperate or subpolar shrubland, (o) distance to coastline, and (p) elevation for glaucous‐winged gulls (green‐dashed line), western gulls (blue‐dotted line), and hybrid gulls (orange‐solid line). Shading indicates 95% confidence intervals calculated across 10 iterations of the models.

We examined how environmental variation is associated with species occurrence data by analyzing the effect of individual variables on model performance using jackknife tests and response curves from our ENMs. The jackknife tests showed that the three most important factors for habitat suitability for glaucous‐winged gulls and hybrid gulls are the same: distance to coastline, mean diurnal range, and elevation. However, these two groups differ in the importance of annual mean temperature and temperature seasonality. In contrast, western gull habitat suitability was largely influenced by temperature seasonality, followed by distance to coastline and elevation (Figure 4). Habitat suitability did not depend strongly on land cover for any of the gull populations (training gain <0.5). The most influential land cover variable was temperate or subpolar grassland for glaucous‐winged gulls, temperate or subpolar needleleaf forests for western gulls, and temperate or subpolar grasslands for hybrid gulls. The response curves produced by our models support the jackknife results. Probability of occurrence for all three gulls is high in low‐elevation habitats close to the coastline, but the relationships between probability of occurrence and environmental variation differ among all three groups for several variables (Figure 5). Glaucous‐winged gulls are more likely to occur in habitats with lower mean diurnal range than western gulls and hybrid gulls. Hybrid gulls prefer habitats with similar temperature seasonality and annual mean temperatures as the glaucous‐winged gulls but have lower habitat breadth. Overall, these results suggest that western gulls, glaucous‐winged gulls, and hybrids occupy different habitats, with greater niche overlap between hybrid gulls and glaucous‐winged gulls.

## 4 DISCUSSION

Our results indicate that environmental gradients within the hybrid zone between western gulls and glaucous‐winged gulls may contribute to maintaining hybrid zone stability under the bounded superiority model. The bounded superiority model predicts both niche divergence between the two parental species and niche divergence between the parental species and the hybrids. Our niche identity and background similarity tests show that western gulls, glaucous‐winged gulls, and their hybrids all occupy significantly different ecological niches, though we did not find evidence for a significant biogeographical barrier. The differences in the response curves and contributions of individual environmental variables suggest that differences in annual mean temperature, temperature seasonality, mean diurnal range, and land cover type may underlie the observed niche divergence.

Under the tension zone model, there should be niche conservation within the hybrid zone among all three gull populations. Our range‐breaking tests found no distinct boundaries between populations. This result disagrees with our niche identity and background similarity test results and could be explained by the complex habitat preferences of gulls (Bell, 1992; Hoffman et al., 1978). Niche diversification between the hybrids and the parental species could be caused by variation in habitat preference along a smoother but more mosaic landscape instead of a steep environmental gradient, which would result in a lack of distinct boundaries between parental species and hybrid distributions detectable by a range‐breaking test. Thus, the range‐breaking test results do not necessarily indicate niche overlap among the three populations. The geographical selection‐gradient model predicts niche divergence between the parental species but not between the hybrids and the parental species (Moore, 1977). We found no support for this model, as the range‐breaking tests suggest a lack of distinct boundaries among the three populations, whereas the niche identity and background similarity tests suggest niche divergence among the three populations.

Our results extend previous work on the nesting ecology of gulls at specific sites in this hybrid zone by explicitly testing habitat associations across the entire region. Though our ENMs suggest that all three populations occupy different ecological niches, we found more niche overlap between hybrid gulls and glaucous‐winged gulls than between hybrid gulls and western gulls for important climatic (temperature seasonality and annual mean temperature) and land cover variables. These results agree with previous work showing that hybrid gulls select similar nesting habitats as glaucous‐winged gulls near the center of the hybrid zone, which is hypothesized to underlie lower nest predation in hybrid gulls compared to western gulls (Good et al., 2000; Hoffman et al., 1978). At northern sites in the hybrid zone, some studies found no differences in nest site selection or reproductive success between hybrid gulls and glaucous‐winged gulls (Megna et al., 2014; Moncrieff et al., 2013). One study found that hybrid gulls had higher hatching and fledging success compared to glaucous‐winged gulls and attributed those changes to similarities in foraging habits between hybrid gulls and western gulls (Good et al., 2000). Our models do not include pelagic areas and therefore cannot detect differences in foraging behavior, but our results do indicate that hybrid gulls occupy a distinct ecological niche from both parental species.

Previous studies of this hybrid zone have searched for evidence of reproductive barriers and found contradicting results (Table 1). Assortative mating based on appearance has been observed within multiple sites in both the parental species and the hybrids, possibly indicating selection against hybrids (Megna et al., 2014, Moncrieff et al., 2013). However, as hybrid gulls display appearances that span a wide spectrum between the two parental species, it can be difficult to interpret the causes and results of this behavior (Bell, 1996, 1997; Good et al., 2000; Hoffman et al., 1978; Megna et al., 2014; Moncrieff et al., 2013). Phenotypic and genotypic cline analyses found both steep, stepped, and more random shape cline models for different features of the gulls, and provided support for either the tension zone model or the bounded superiority model (Bell, 1996; Gay et al., 2008). The hybrid zone has also been found to be asymmetrical, with more introgressed individuals found north of the center of the zone (Gay et al., 2008). This geographic shift could be due to movement in the hybrid zone or differences in selection of hybrids in different regions of the hybrid zone. We found evidence supporting more similar habitat preferences between hybrid gulls and glaucous‐winged gulls than between hybrid gulls and western gulls, which may contribute to the observed asymmetry in the hybrid zone. Overall, the niche differences between all three groups observed in this study, combined with previous evidence of high hybrid reproductive success and density within the hybrid zone, provide support for the bounded superiority model.

There are several challenges inherent to predicting ecological niches that we aimed to address in this study. The presence‐background nature of Maxent models may limit our ability to predict unsuitable habitats (e.g., Jiménez‐Valverde et al., 2008; Svenning & Skov, 2004). However, part of the information contained in absence data is also available in presence data, and presence–absence modeling avoids the problem of false absences, which can be more detrimental to niche prediction (Elith et al., 2011). We acknowledge that identifying gull species in the field can be challenging, and it is possible that the eBird occurrence data included identification errors. We validated our models by comparing the transferability of our ENMs trained on the entire dataset with models trained on the much smaller set of records that have been reviewed by experts through eBird or have associated confirmed breeding codes. In addition, we used species occurrence data for the two parental species from another data source, the North American Breeding Bird Survey (BBS), and performed a model transferability test to check if ENMs are consistent across different sources (Appendix S1). The resulting high AUC values for these analyses (>0.75 for all tests; Appendix S1) give us confidence in the accuracy of our Maxent models. Our analyses consider the comparatively larger ranges of the parental species outside of the hybrid zone, which could have influenced the accuracy of our models in predicting the ecological niches of the parental individuals within the current known hybrid zone (Lee‐Yaw et al., 2016). However, as gulls can travel long distances within days, only considering individuals within or near the hybrid zone would have influenced the accuracy of niche estimation. Finally, our models only included abiotic environmental variables. Biotic interactions can be highly influential on species ranges but are challenging to incorporate given the large number of possible interactions between species (Paquette & Hargreaves, 2021). Future studies are needed to characterize the potential influence of biotic factors on the stability of this hybrid zone.

Our study provides additional support for the importance of exogenous selection in maintaining the hybrid zone between glaucous‐winged gulls and western gulls, with selection favoring hybrids over either parental species. We now know that hybrid fitness can be quite variable, and understanding the factors that affect hybrid performance is critical for predicting the evolutionary dynamics of hybrid zones (Curry, 2015; Muraro et al., 2022). The tension zone model permits hybrid zone movement in response to dispersal of the parental species, while the bounded superiority model predicts that hybrid zones will be stationary as long as the environmental gradient across the hybrid zone persists (Curry, 2015; Key, 1968; Moore, 1977). More broadly, the type (endogenous or exogenous) and direction (for or against hybrids) of selection shape patterns of fitness across space, which is crucial for studying the evolution of reproductive isolation and predicting how environmental change may affect patterns of biodiversity (Curry, 2015; Wang et al., 2022).

## 5 CONCLUSION

Our study provides additional insights into the role of environmental variation in maintaining the western × glaucous‐winged gull hybrid zone. Using ENMs fitted with data from citizen science databases, we characterized the ecological niches of the parental species and the hybrids and showed that all three populations occupy different niches. Our results best support the bounded superiority model, suggesting that hybrid gulls are better adapted to the environment in the hybrid zone than either of the two parental species, which aligns with previous studies that observed higher reproductive success of hybrid individuals. Additional empirical support for the bounded superiority model indicates the potential importance of environmental variation in the maintenance of hybrid zone stability.

## AUTHOR CONTRIBUTIONS

**Xuewen Geng:** Conceptualization (lead); data curation (lead); formal analysis (lead); methodology (lead); visualization (lead); writing – original draft (lead); writing – review and editing (equal). **Jeremy Summers:** Conceptualization (equal); methodology (equal); supervision (equal); visualization (supporting); writing – review and editing (equal). **Nancy Chen:** Conceptualization (equal); funding acquisition (lead); methodology (equal); project administration (lead); supervision (lead); visualization (supporting); writing – review and editing (equal).

## CONFLICT OF INTEREST STATEMENT

The authors declare no competing interests that could influence the work in this paper.

## Supporting information


Data S1

## Data Availability

All metadata and code used in this study are available on GitHub: https://github.com/ggg80/HybridGull_Repo.
