# Supplementary material for: Ecological niche contributes to the persistence of the western × glaucous‐winged gull hybrid zone
Source: Ecol Evol. 2024 Jul 11;14(7):e11678. doi: 10.1002/ece3.11678 (PMC11239321; doi:10.1002/ece3.11678)
Supplement: Supplementary file 1 — Data S1 [file ECE3-14-e11678-s001.pdf]

Supplementary Figures

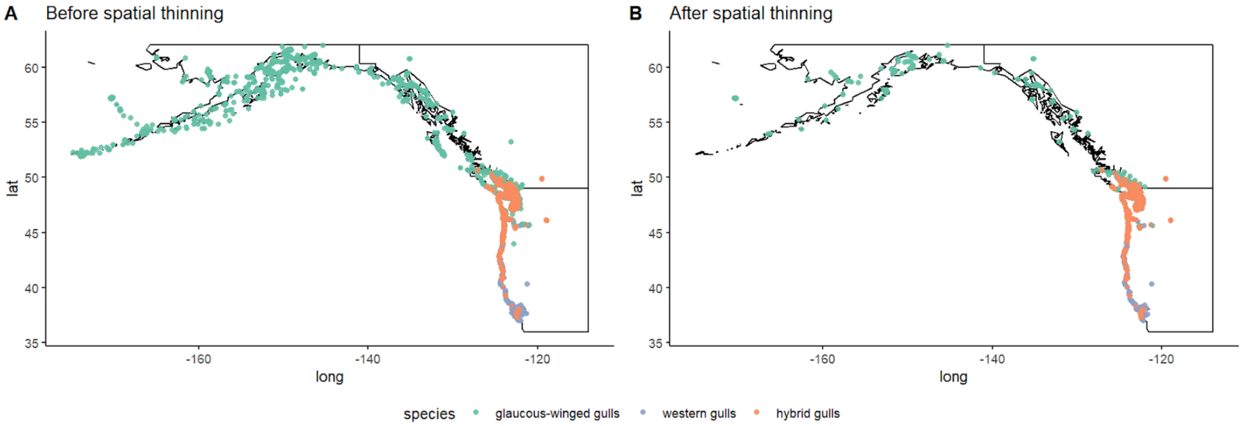

Figure S1: Species occurrence points for glaucous-winged gulls (green), western gulls (blue), and hybrid gulls (orange) (A) before and (B) after spatial thinning.

Appendix 1. Model Transferability Test

Species identification

As gulls are notorious for their physical similarities across species and challenging species identification, we validated our eBird environmental niche models (ENMs) using model transferability tests (Sequeira et al. 2018; Yates et al. 2018)with eBird records that have been validated and reviewed as well as species occurrence data from another citizen science database, the American Breeding Bird Survey (BBS; (Ziolkowski Jr et al. 2022). Model transferability refers to the ability of a model to predict reliable results in unsampled geographical or temporal regions, or on different datasets (Peterson et al. 2007). We used the evaluate() function from the maxent package in R to calculate the area under the receiver operating curve (AUC) value for models built on the eBird databases with the relevant testing dataset (Jurka 2012). We also built another set of ENMs based on either validated records or BBS data and tested their ability to predict species occurrences in our eBird dataset.

A subset of the eBird species occurrence records included rich media or further details and were manually validated by an expert eBird reviewer. In our study extent, we found 90 validated and reviewed records for glaucous-winged gulls, 51 for western gulls, and 39 for hybrid gulls. We removed these records from our training dataset and constructed ENMs as before (see Materials and Methods), then performed model transferability tests for each group. Resulting AUC values were high for all three groups (0.748 for glaucous-winged gulls, 0.770 for western gulls, and 0.805 for hybrid gulls). ENMs constructed with validated records and tested on non-validated eBird records also had high transferability for all three groups (AUC = 0.812 for glaucous-winged gulls, 0.83 for western gulls, and 0.756 for hybrid gulls).

BBS is a long-term breeding bird population and distribution monitoring program that records species occurrence and abundance along more than 4000 routes each year by bird experts (Ziolkowski Jr et al. 2022). We extracted occurrence data from the same study extent using the same filters as before and constructed Maxent models using the same settings as the eBird data (see Materials and Methods). Our filtered BBS dataset included 226 observations of glaucous-winged gulls, 78 observations of western gulls, and 5 observations of hybrid individuals. We did not have enough hybrid occurrence records in the

BBS dataset to reasonably test model transferability, so we only considered the two parental species.The resulting AUC values between each pair of parental species models are high: models constructed with eBird data and tested on BBS data had AUCs of 0.941 for glaucous-winged gulls and 0.967 for western gulls, and models constructed with BBS data and tested on eBird data had AUCs of 0.879 for glaucous-winged gulls and 0.936 for western gulls, which suggests that our models are consistent between different databases. Together, these results suggest that species identification in eBird data is sufficiently accurate to construct valid ENMs, and we use the models constructed from the larger, filtered eBird dataset in downstream analyses.

Breeding Individuals

As non-breeding gulls may occupy different habitats from breeding gulls even during the breeding season, we used model transferability tests to confirm that our models are accurately capturing the environmental niches of breeding birds. eBird allows observers to document specific behaviors by tagging observations with breeding codes, though not all users report breeding codes. We filtered our species occurrence data for records with a breeding code associated with confirmed breeding: carrying food (CF), recently fledged young (FL), feeding young (FY), nest with eggs (NE), nest with young (NY), and occupied nest (ON). After filtering, we had 240 observations of glaucous-winged gulls, 321 observations of western gulls, and 23 observations of hybrid gulls. We constructed ENMs using eBird records without associated breeding codes and tested their performance with records with confirmed breeding codes. We also performed backward model transferability tests by fitting models using records with confirmed breeding codes and testing model performance on the larger dataset of records without associated breeding codes. Both model transferability tests resulted in high AUC values for all three groups (0.904 and 0.873 for glaucous-winged gulls; 0.961 and 0.950 for western gulls, and 0.937 and 0.848 for hybrid gulls, respectively). Our results suggest that our eBird models trained on data filtered for the breeding season (but not for confirmed breeding codes) accurately capture the environmental niches of breeding individuals. As not all eBird users record breeding codes, we used models trained on our original eBird dataset in downstream analyses.

Appendix 2. Code

Please refer to this GitHub repository for all code used in this manuscript: https://github.com/ggg80/HybridGull_Repo

References

Jurka, T.P. (2012) 'maxent: An R Package for Low-memory Multinomial Logistic Regression with Support for Semi-automated Text Classification', *The R Journal*, 4(1), 56, available: http://dx.doi.org/10.32614/RJ-2012-007.

Peterson, A.T., Papeş, M. and Eaton, M. (2007) 'Transferability and model evaluation in ecological niche modeling: a comparison of GARP and Maxent', *Ecography*, 30(4), 550-560, available: http://dx.doi.org/10.1111/j.0906-7590.2007.05102.x.

Sequeira, A.M.M., Bouchet, P.J., Yates, K.L., Mengersen, K. and Caley, M.J. (2018) 'Transferring biodiversity models for conservation: Opportunities and challenges', *Methods in Ecology and Evolution*, 9(5), 1250-1264, available: http://dx.doi.org/10.1111/2041-210X.12998.

Yates, K.L., Bouchet, P.J., Caley, M.J., Mengersen, K., Randin, C.F., Parnell, S., Fielding, A.H., Bamford, A.J., Ban, S., Barbosa, A.M., Dormann, C.F., Elith, J., Embling, C.B., Ervin, G.N., Fisher, R., Gould, S., Graf, R.F., Gregr, E.J., Halpin, P.N., Heikkinen, R.K., Heinänen, S., Jones, A.R., Krishnakumar, P.K., Lauria, V., Lozano-Montes, H., Mannocci, L., Mellin, C., Mesgaran, M.B., Moreno-Amat, E., Mormede, S., Novaczek, E., Oppel, S., Ortuño Crespo, G., Peterson, A.T., Rapacciuolo, G., Roberts, J.J., Ross, R.E., Scales, K.L., Schoeman, D., Snelgrove, P., Sundblad, G., Thuiller, W., Torres, L.G., Verbruggen, H., Wang, L., Wenger, S., Whittingham, M.J., Zharikov, Y., Zurell, D. and Sequeira, A.M.M. (2018) 'Outstanding Challenges in the Transferability of Ecological Models', *Trends in Ecology & Evolution*, 33(10), 790-802, available: http://dx.doi.org/10.1016/j.tree.2018.08.001.

Ziolkowski Jr, D., Lutmerding, M., Aponte, V. and Hudson, M.-A. (2022) *2022 Release - North American Breeding Bird Survey Dataset (1966-2021)* [dataset], available: 10.5066/P97WAZE5.
